# *Clostridium septicum*-induced gangrene in the right lower extremity complicating pneumatosis in the right ventricle and the pulmonary artery and occlusion of right femoral artery: a case report

**DOI:** 10.1186/s12879-021-06653-z

**Published:** 2021-09-16

**Authors:** Hui-Dan Jing, Lei Li, Jun-Ying Tian, Dong-Po Jiang

**Affiliations:** 1 Department of Intensive Care Unit, Daping Hospital, Army Medical University, Chongqing, 400042 China; 2 Department of Trauma Center, Daping Hospital, Army Medical University, Chongqing, 400042 China; 3 Department of Medical English, College of Foreign Languages, Chongqing Medical University, Chongqing, 400016 China

**Keywords:** *Clostridium septicum*, Gas gangrene, Air embolism, Artery occlusion, Case report

## Abstract

**Background:**

Gas gangrene is usually manifested as myonecrosis and subcutaneous gas accumulation, but rarely manifested as arterial occlusion or pneumatosis in the right ventricle and the pulmonary artery.

**Case presentation:**

We report a case of gas gangrene caused by *Clostridium septicum*. The patient developed gas gangrene after being pecked by a chicken but turned for the better following antibiotic treatment and debriment. Imaging test revealed a rare occlusion of the right femoral artery and pneumatosis in the right ventricle and the main pulmonary artery.

**Conclusions:**

In the presence of gas gangrene, special care must be taken to prevent against the formation of circulatory air embolism. The gas gangrene-induced gangrene in the limb of this patient might be attributed to the combined action of infection and arterial occlusion. MDT (Multidisciplinary team)-Green Channel mode is conductive to treatment success of gas gangrene.

## Background

Gas gangrene is an acute, fatal and specific infection with rapid progression and poor prognosis. With low mortality rate but high disabliity rate, its mortality rate is as high as 20–40% [1, 2]. Gas gangrene is a highly lethal infection of soft tissue characterized by myonecrosis and subcutaneous pneumatosis [3]. In rare cases, it may cause gangrenous infection of internal organs such as liver [4], spleen [5, 6], and bladder [7]. Here, we report the rare case of *Clostridium septicum*-induced gas gangrene in the right lower limb complicating pneumatosis in the right ventricle and the pulmonary artery and occlusion of the right femoral artery.

## Case presentation

An 85-year-old male farmer presented a minor oozing wound after being pecked by a chicken on his right heel while raising poultry 2 days before admission. The wound was disinfected with alcohol. 8 h later, the patient visited the community hospital again due to the aggravation of wound pain. It was noted from the medical record of community hospital that the wound was small without redness and swelling and the patient was treated with wound disinfection, tetanus immunoglobulin injection and oral cephalosporin. The next day, the patient was referred to the district hospital for emergency treatment due to the progressive aggravation of wound pain in the right lower limb. No obvious abnormality was noted in his wound and right lower limb at that time. In the district hospital, the patient was diagnosed as mild wound infection, which was treated with intravenous cephalosporin for infection control and morphine for pain relief. However, the pain was intensified and began spreading throughout the body gradually. The swelling at the chicken-pecking site remained stable. The patient became anxious and irritated due to pain aggravation. Three hours prior to his admission, he presented with drowsiness, increased heart rate and scattered small blisters on his right lower limb.

**Fig. 1:**
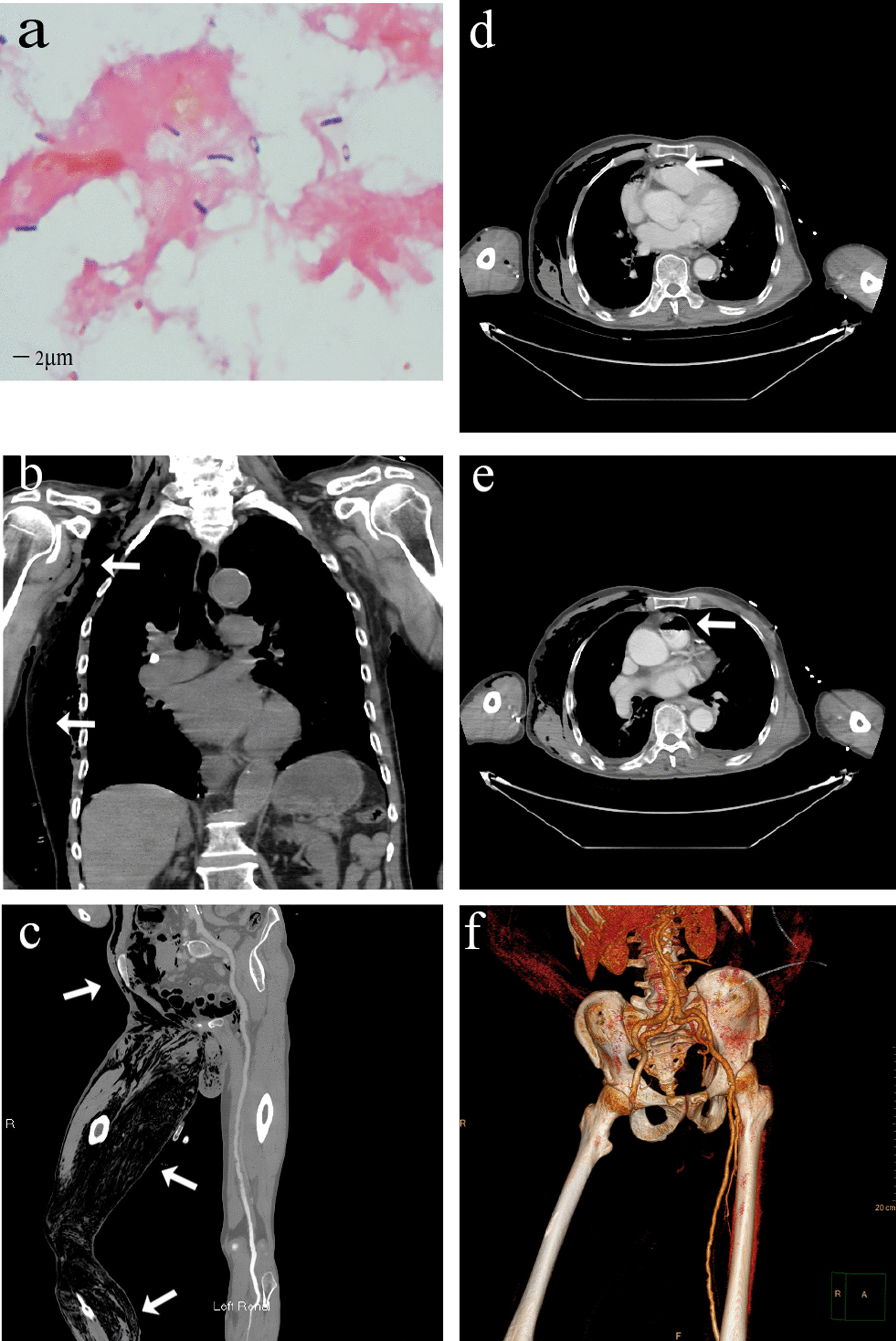
Bacterial smear and CT images. **a** Gram-positive bacilli on bacterial smear. **b** Pneumatosis in the right side of the neck, chest wall, upper abdomen (shown by the white arrow). **c** Pneumatosis in the right lower abdomen and lower extremities (shown by the white arrow). **d** Pneumatosis in the right ventricle (shown by the white arrow). **e** Pneumatosis in the pulmonary artery (shown by the white arrow). **f** CTA reconstruction shows occlusion of the right femoral artery. *CT* computerized tomography, *CTA* computerized tomography angiography

After arrival at our hospital, he was transferred to emergency department immediately. On physical examination, the patient was extremely weak and exhausted with a body temperature of 38.4 °C, heart rate of 120 beats/min, blood pressure of 151/90 mmHg, and respiration rate of 25/min. He presented with extensive subcutaneous crepitus on right torso, cyanosis in multiple sites of skin, scattered blood blisters, scattered exfoliative dermatitis, a serosanguinous drainage with foul-smelling odor, swelling in the right lower limb, paleness and coldness in right foot, non-pulsation in dorsalis pedis, and 2 × 2.5 cm blood blisters in the right heel. Gram-positive bulky bacilli were identified from the smear of the subcutaneous interstitial fluid in the right lower limb (Fig. 1a). Fusobacterium infection was suspected to be responsible for gas gangrene so that clindamycin was administered empirically. Emergent whole-body computerized tomography (CT) and CT angiography (CTA) revealed multiple pneumatosis in the right side of body (Fig. 1b, c), the right ventricle and the pulmonary artery (Fig. 1d–e,) and occlusion in right femoral artery (Fig. 1f). One hour later, the patient was sent to the operating room for surgical debridement of the right chest wall, right lower limb and perineum. Intraoperatively, the patient developed septic shock.

Postoperatively, the patient was transferred to the intensive care unit (ICU) and maintained on ventilator and treated with analgesics, sedatives, and continuous renal replacement therapy (CRRT) etc. Clindamycin and meropenem were administered in combination prophylactically to prevent against mixed infection. The patient showed gradual stability in organ function and improvement in microcirculation perfusion after treatment (Table 1). The vasoactive drug was discontinued 24 h after surgery. VITEK^®^ MS system was applied for rapid bacterial identification and the identification results showed that *Clostridium septicum* was positive in the exudates but negative in blood. Given the extensive myonecrosis in the right lower limb and the total occlusion of the right femoral artery, secondary amputation surgery was planned for this patient. Regrettably, a decision to withdraw from aggressive treatment was made by the family members because they thought that the “incomplete” body following limb amputation is against their local customs and tradition. The patient died the next day after leaving the hospital.

**Table 1:** Blood tests during the course of the disease

	Day 1 (district hospital)	Day 2 (emergency department)	Day 2 (ICU)	Day 3 (ICU)
RBC (× 10^12^/L, 4.3–5.8)		3.56	2.56	2.28
Hb (g/L, 130–175)		114	82	73
Platelets (× 10^9^/L, 94–268)		78	35	19
WBC (× 10^9^/L, 3.5–9.5)	5.75	7.40	4.36	3.44
Neutrophils (× 10^9^/L, 1.8–6.3)	5.02	2.40	3.48	2.79
Lymphocyte (× 10^9^/L, 0.8–4.0)		0.25	0.51	0.36
Procalcitonin (ng/mL, < 0.25)		> 100	> 100	> 100
CRP (mL/L, 0–8)	149	163	154	196
IL-6 (pg/mL, < 3.4)	> 5000	> 5000	> 5000	> 5000
PT (s, 9.4–13.8)		13.8	14.5	14.9
Fibrinogen (g/L, 2–4)		3.40	3.67	3.02
d-dimer (ug/L, 0–232)		10,931	8191	1252
R (min, 5–10)			8.7	12
K (min, 1–3)			2.8	2.3
MA (mm, 50–70)			56.2	58.3
CI (− 3 to 3)			− 3.5	− 5
LY30 (%, 0–8)			0	0.3
Total bilirubin (mg/dL, 0.1–1.4)		31.9	16.4	30.3
Direct bilirubin (mg/dL, 0–0.5)		14.4	7.9	17.7
Indirect bilirubin (mg/dL, 0–0.9)		17.5	8.5	12.6
Creatinine (µmol/L, 57–111)		205	120	83
Myohemoglobin (ug/L, 25–72)		> 3000	> 3000	> 3000
Creatine kinase (U/L, 38–174)			19,183	9220
BNP (pg/mL, < 100)		2907	3041	2568
Glucose (mmol/L)			9.8	8.9
pH, arterial		7.31	7.29	7.45
PO_2_/FiO_2_		312	306	331
Lactate (mmol/L, < 2 mmol/L)		3.1	4.9	3.8

*ICU* intensive care unit, *RBC* red blood cell, *Hb* hemoglobin, *WBC* white blood cell, *PT* prothrombin time, *CRP* c-reactive protein, *R* coagulation response time, *K* the time required from the end point of R to reach the mark range of 20 mm, *MA* maximum amplitude, *CI* comprehensive clotting index, *LY30* the rate at which the clot amplitude decreased within 30 min after the MA value was determined was measured, *BNP* brain natriuretic peptide

## Discussion and conclusion

Gas gangrene, or clostridial myonecrosis, is a severe acute specific anaerobic bacterial infection caused by *Clostridium perfringens *[2, 5]*. Clostridium* infection may be secondary to a major trauma or occur either in the presence or absence of mucocutaneous damage [6, 8]. In this patient, severe pain was a warning sign of a serious infection. CT or MRI can usually reveal the presence of gas accumulation at the site of infection [9]. Imaging test of this case showed signs of pneumatosis in the right ventricle and the main pulmonary artery and occlusion of the right femoral artery. To our knowledge, this is the first report of gas gangrene case with these radiographic signs.

### Inconformity between pain and trauma degree

Gas gangrene is likely to be misdiagnosed due to the unremarkable early wound reaction [10]. This rare gas gangrene case started with local pain and minor skin lesion in the absence of remarkable early redness and swelling. In his first two visits of hospital, diagnosis of gas gangrene was not confirmed, however, the infection rapidly spread along his right side of body within 48 h accompanied by shock and multiple organ dysfunction syndrome (MODS), indicating that severe gas gangrene may also arise from minor trauma. Therefore, special medical care must be taken to prevent against gas gangrene if the post-traumatic sharp pain is inconsistent with the wound surface reaction.

### Imaging findings: circulatory pneumatosis and arterial occlusion

The exact origin of pneumatosis in right ventricle and the main pulmonary artery remains unknown. At the time of CT examination, the patient had only one indwelling needle in the peripheral vein, and the iatrogenic procedure-induced intravenous pneumatosis was less likely to occur. The negativity of bacteria in blood culture excluded the possibility of gas production by circulating bacteria. The gas might come from extravascular tissue. Infection with *Clostridium septicum* or other gas-producing bacteria may lead to tissue decomposition and overproduction of gas. Moreover, the vascular endothelial cell damage following infection may lead to increase in vascular permeability [11]. Due to the relatively low pressure in the venous system, gas is likely to enter into the right heart and accumulate there. When the patient assumes a supine position, the right ventricle and the main pulmonary aorta were in a relatively high position, allowing gas accumulation there. The gas entry into the pulmonary artery may lead to pulmonary artery embolism. The patient with gas gangrene is susceptible to gas embolism, and the extensive embolization may lead to sudden death [12]. However, debridement and reduction of tissue pressure may contribute to a lower risk of gas embolism.

The underlying mechanism of right femoral artery occlusion remains unclear as well. The patient denied discomfort in his right lower limb prior to onset of symptoms. CTA revealed a maximum arterial stenosis of 30% in the lower limb and multiple artery plaques. Evidently, the occlusion of the right femoral artery was caused by infection. The possible mechanisms of right femoral artery occlusion include the following: (1) Acute compartment syndrome: extensive myonecrosis and gas accumulation in subcutaneous soft tissue on the right side of the body resulted in acute compartment syndrome in the right lower limb; (2) Thrombosis: thrombus formation may be trigged by pathogenic bacteria and inflammatory mediators in the presence of sepsis through multiple pathways like up-regulation of procoagulant pathway, down-regulation of physiological anticoagulant production, and inhibition of fibrin decomposition, etc. [7]. During the course of disease, blood clotting was monitored closely, with d-dimer showing a significant increase, suggesting a high possibility of thrombosis; (3) Sepsis-induced impairment of vascular tone [13]; (4) Vascular diseases (e.g. multiple atherosclerosis). Gas gangrene-induced limb gangrene might be caused by the combined action of infection and vascular occlusion. In this case, the right femoral artery occlusion aggravated the gangrene of the right lower limb, justifying the need for a secondary amputation surgery. The artery occlusion may be a self-protective response of the body to prevent the infection from spreading throughout the body. The underlying mechanisms for artery occlusion remain explored further.

### Reflections on the treatment of gas gangrene

Gas gangrene progresses rapidly, however, timely treatment is the key for an improved prognosis [14]. Once gas gangrene infection is suspected, MDT-Green Channel mode should be initiated for achieving rapid diagnosis and treatment plan through the collaboration of multidisplinary personnel [15, 16]. In our report, this patient turned for the better in overall condition due to the application of the MDT-green channel mode.

Some problems in the treatment of this patient warrant further discussion. First, how can an early diagnosis of gas gangrene be achieved prior to the presence of characteristic symptoms? The presence of severe pain in minor wound may be an important indicator. Early bacterial identification of the wound exudate by smear or by more rapid and sensitive gene-sequencing techniques may contribute to early confirmed diagnosis [17]. Second, regarding treatment of circulatory pneumatosis, given the patient's stability in respiration and circulation after treatment, preventative measures aiming at circulatory pneumatosis including cardiac puncture to withdraw air was not performed. However, it is too late if cardiac puncture performed after presence of pulmonary embolism. Hence, the timing of intervention remains to be further explored. Third, due to the severe myonecrosis, vascular intervention aiming at clarifying the cause of vascular occlusion and achieving recanalization was not attempted. In patients with  confined myonecrosis, infection control, and stable circulation, interventional recanalization efforts may help reduce the risk of undergoing amputation surgery. Fourth, in this case, the lymphocyte count showed a significant reduction, excluding the possibility of presence of immunodeficiency diseases. Evidently, this is sepsis-induced immunosuppression [18]. The timing of immunoregulation administration remains to be explored further in future study.

## Data Availability

The datasets used and/or analyzed during the current study are available from the corresponding author on reasonable request.

## Abbreviations

MDT: Multidisciplinary team
CT: Computerized tomography
CTA: Computerized tomography angiography
ICU: Intensive care unit
CRRT: Continuous renal replacement therapy
MODS: Multiple organ dysfunction syndrome
PCT: Procalcitonin
IL-6: Interleukin-6
CRP: C-reactive protein
